# Cisternal, vascular, and parenchymal landmarks in amygdalohippocampectomy for mesial temporal sclerosis: an index case with learnings from 820 resections

**DOI:** 10.3171/2024.4.FOCVID2428

**Published:** 2024-07-01

**Authors:** Akhade Bhushan Sadashiv, Lokesh Vellore Dasarathan, George Chandy Vilanilam, Sam Scaria, Krishnakumar Kesavapisharady, Easwer Hariharan Venkat

**Affiliations:** Department of Neurosurgery, Sree Chitra Tirunal Institute for Medical Sciences and Technology, Thiruvananthapuram, Kerala, India

**Keywords:** mesial temporal lobe, amygdala, hippocampus, amygdalo-hippocampectomy

## Abstract

Cortico-amygdalo-hippocampectomy is the most common epilepsy surgery resection in adults and offers excellent outcomes. Seizure outcome benefits range from 75% to 88% with a 2%–4% adverse event rate. The safety profile and outcomes could be enhanced further by clearly defining key surgical landmarks that could also aid tumoral resections in the mesial temporal lobe and selective mesial resections. The authors present their learnings of intraoperative landmarks (cisternal, parenchymal, and vascular) and surgical substeps through an index case of cortico-amygdalo-hippocampectomy with lessons from 820 resections.

The video can be found here: https://stream.cadmore.media/r10.3171/2024.4.FOCVID2428

## Transcript

We present the cisternal, vascular, and parenchymal landmarks in amygdalo-hippocampectomy for mesial temporal sclerosis through an index case, with learnings from 820 resections.

### 0:35 Clinical Features.

Our index case was a 32-year-old male, right-handed, with history of febrile seizures and 20 years’ duration of habitual hypomotor seizures. He was not responding to three anticonvulsant medications, and a presurgical workup with a video-EEG showed left temporal dysrhythmic spikes.

### 0:54 Imaging Features.

MRI showed left hippocampal sclerosis and left temporal neocortical atrophy. There was a T2 FLAIR hyperintensity in the left hippocampus with reduced volume and a delayed T2 relaxometry. The arterial spin labeling showed left temporal and mesial temporal hypoperfusion.

### 1:16 Plan and Surgical Steps.

He was thus planned for a left anterior temporal lobectomy and amygdalo-hippocampectomy.^1–3^ In the supine position, the head was fixed on a Mayfield three-pin clamp with 15° extension and 20° rotation. A left temporal question mark flap was made. A left temporal craniotomy was done. The dura was opened based on the sphenoid ridge. Intraoperative electrocorticography (ECoG) was done using a 20-contact grid, and left temporal spikes were seen. The limits of resection were marked from the pole 4 cm, this being a dominant temporal resection. On the nondominant side, we prefer about 5-cm resections.

### 2:03

The pia-arachnoid was then gently coagulated. The gray and the white matter was dissected maintaining an angulation of the bipolar just lateral to the edge of the tent. The superior, middle, inferior, and the lateral occipito-temporal gyrus was resected as part of an en masse resection. The temporal horn was then dissected open gently. The landmarks used were 3 cm from the pole, at a depth of 3 cm, at the level of the middle temporal gyrus. The hippocampus, amygdala, and the medial temporal structures were then identified.^1,2^

### 2:44

Depth electrode was placed for ECoG recordings from the head of the hippocampus and spikes were seen in contacts 1 and 2. Next, the depth electrode recordings were obtained from the amygdala. Anteromedial to the hippocampal head is the amygdala. The limits of the amygdala resection were identified. The mesial limit is up to the pia of the ambient cistern. The superior limit up to the optic tract and the medial-posterior limit is the inferior choroidal point.^4^ The Wen’s line, an imaginary line from the ICA bifurcation to the inferior choroidal point, was used as a landmark and the resection was done inferolateral to it.^5^ The amygdala was resected in a piecemeal manner using suction. Next, the temporal horn was dissected further open to expose the entire length of the hippocampus (the head, body, and part of the tail).

### 3:45

The collateral sulcus was then incised and the lateral basal lobe lateral to the collateral sulcus was resected en masse. This resection was extended right up to the pia-arachnoid at the temporal base. Next, the choroidal fissure was dissected open carefully with gentle pressure on the hippocampus, the specimen side. Under high zoom, the choroidal fissure was gently dissected and the cisternal structures, the PCA-PCOM (posterior cerebral artery-posterior communicating) complex, the mesencephalon, and the basal vein of Rosenthal were seen. The choroidal fissure was further dissected open to evert the head of the hippocampus so that the P2 segment of the PCA could be seen clearly winding around the brainstem.

### 4:42

The taenia fimbriae of the fornix was patiently dissected, avoiding any blind coagulation in the choroidal fissure. The hippocampus head was thus mobilized further and the medial disconnection of the hippocampus on the way to an en masse resection was done. The hippocampus head was gently visualized, and the PCA along with its branches, the hippocampal artery, were seen. The hippocampus head was gently everted in order to ensure a complete en masse resection, and the hippocampal sulcus was now seen with the hippocampal artery and its branches both to the hippocampus and the parahippocampal gyrus. The hippocampal vessels were gently coagulated close to the specimen side and the pia of the hippocampal sulcus was incised. Further on, the head of the hippocampus was subpially resected and this subpial resection was extended further into the anterior parahippocampal gyrus and this subpial resection was further on extended posteriorly to the mid- and posterior parahippocampal gyrus.^6^ Next, the posterior disconnection of the hippocampus was done along with the lateral disconnection subpially to ensure that an en masse resection of the hippocampus was done. The posterior disconnection was done at the level of the lateral mesencephalic sulcus to involve a part of the hippocampus tail as well.

### 6:25

About 3.5 cm of the hippocampus was resected en masse including the head, body, and part of the tail of the hippocampus just short of the calcar avis. The remaining parahippocampal gyrus stump was also further disconnected posteriorly, and next the uncus was resected subpially to see the tentorial edge and the cisternal structures, the oculomotor nerve, and the PCOM-PCA complex. This is the bed of the resection. And this is the post-resection ECoG showing reduced spikes. No additional tailoring of the resection was done based on the postoperative ECoG. These are the resection limits as seen on the postoperative MRI. The patient did well, with an Engel class I seizure outcome and no neurological deficits.

### 7:33 Discussion.

Next, we discuss key aspects, like avoiding an anterior choroidal artery injury. Gentle dissection of the choroidal fissure, maintaining the subpial plane during uncal and amygdala resection, and avoiding blind coagulation are important. A word of caution about venous anomalies like an anteriorly placed vein of Labbé and a veinless sylvian fissure with a dominant anterior temporal vein. These need to be preserved with an intervascular resection.

8:32 The key parenchymal, cisternal and vascular landmarks include the oculomotor nerve, the temporal horn with the intraventricular contents, the posterior cerebral artery, and the anterior choroidal artery.^1,2^ Here is a quick view of these landmarks as seen in intraoperative snapshots: the hippocampal artery, a schematic view of the resection, the anterior choroidal artery at the inferior choroidal point, the posterior cerebral artery after everting the hippocampal head, the basal vein of Rosenthal in the ambient cistern, the mesencephalon, the oculomotor nerve beyond the tentorial edge, the basilar artery, and the fourth cranial nerve.^5^

### 8:54

This is the edge of the tentorium with the limits of resection and a chronic uncal herniation, which is usually seen. The choroidal fissure, the hippocampal sulcus on an eversion of the hippocampal head, the collateral eminence at the floor of the temporal horn, the parahippocampal gyrus, and the dentate gyrus. The stria terminalis may occasionally be seen. This is the description of the Wen’s line, inferolateral to which lies the resection of the amygdala.^5^ Wide variations exist in the lie of the Meyer’s loop. However, resections over 3.5 cm from the pole risk disruption of the Meyer’s loop, causing a quadrantanopia.^7–9^ Hence, large neocortical resections extending beyond the medial aspect of the roof of the temporal horn may be avoided.^10^ These landmarks are also useful for selective amygdalo-hippocampectomies.^6^

### 9:55 Conclusions.

To conclude, key cisternal, vascular, and parenchymal landmarks serve as "lighthouses" in a sea of brain parenchyma in the temporal lobe. Cortico-amygdalo-hippocampectomy limits and landmarks ensure safety and adequacy of the procedure.

## Disclosures

The authors report no conflict of interest concerning the materials or methods used in this study or the findings specified in this publication.

## Author Contributions

Primary surgeon: Vilanilam. Assistant surgeon: Sadashiv, Dasarathan, Scaria. Editing and drafting the video and abstract: Vilanilam, Sadashiv, Dasarathan. Critically revising the work: Vilanilam, Sadashiv, Dasarathan, Venkat. Reviewed submitted version of the work: Vilanilam, Sadashiv, Dasarathan, Kesavapisharady. Approved the final version of the work on behalf of all authors: Vilanilam. Supervision: Vilanilam.

## Supplemental Information

### Patient Informed Consent

The necessary patient informed consent was obtained in this study.
